# Supplementary material for: Natural and synthetic antimicrobials reduce adherence of enteroaggregative and enterohemorrhagic Escherichia coli to epithelial cells
Source: PLoS One. 2021 May 3;16(5):e0251096. doi: 10.1371/journal.pone.0251096 (PMC8092791; doi:10.1371/journal.pone.0251096)
Supplement: S1 Fig — (DOCX) [file pone.0251096.s001.docx]

S1 Fig. Experiments to determine the efficacy of subinhibitory concentrations of compounds/extracts to inhibit *E. coli* adhesion.


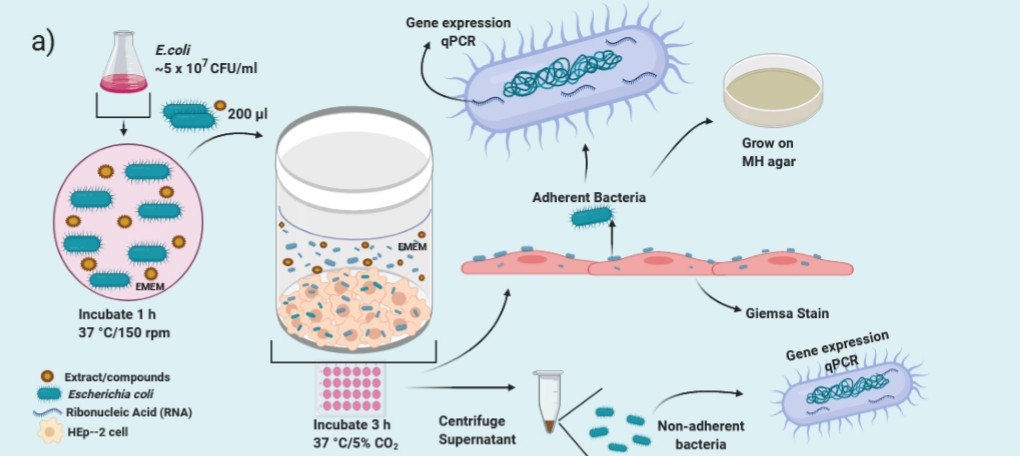


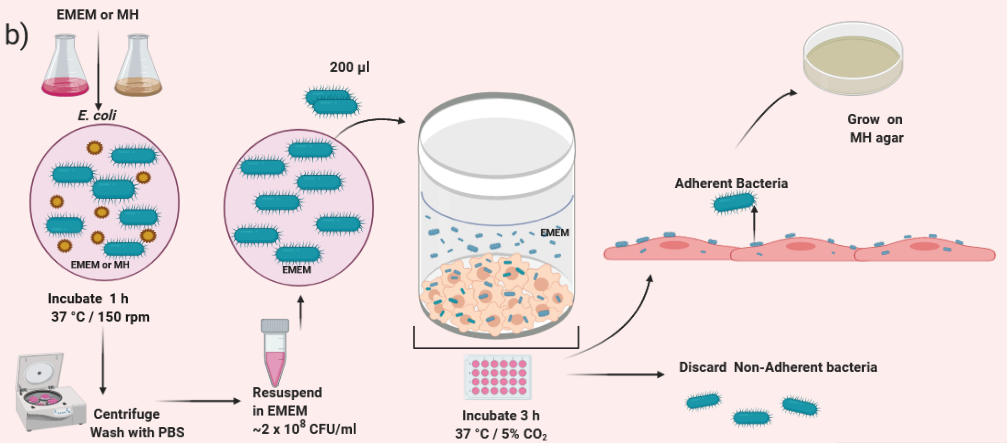


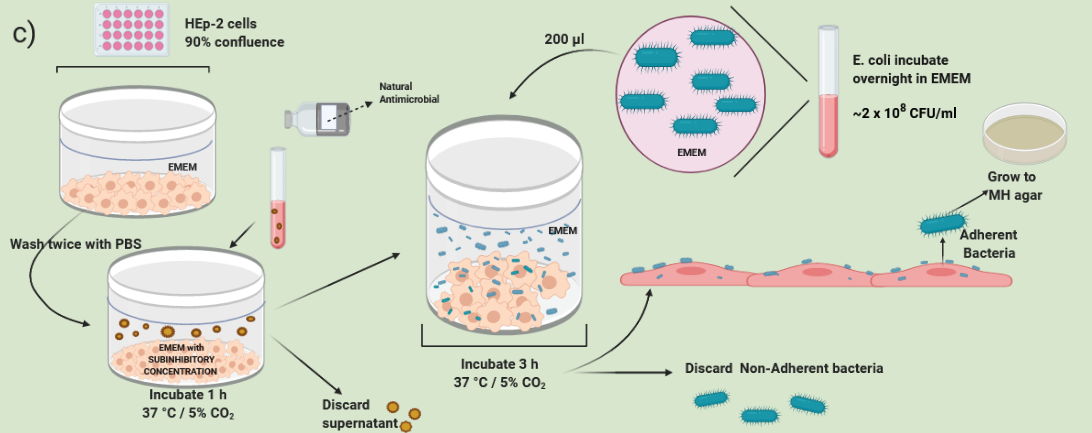


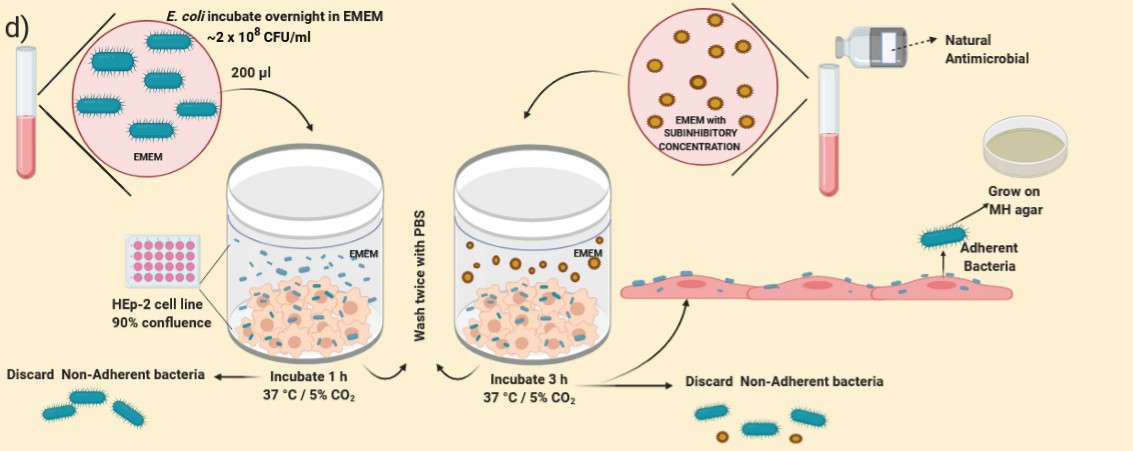


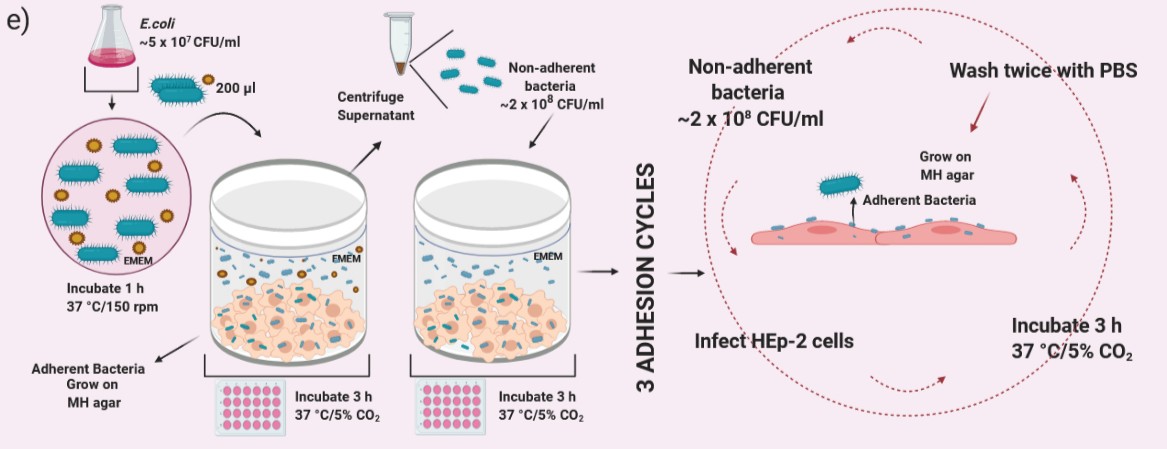


a) *E. coli* pre-incubated in EMEM for 1 h with Sub-MBC of antimicrobials were used to infect confluent monolayer of HEp-2 cells (100:1 *E. coli*:HEp-2), cells were washed after 3 h and *E. coli* adhered was determined in Mueller Hinton agar.

b) *E. coli* were pre-incubated in EMEM (1) and MH broth (2) plus Sub-MBC antimicrobials. After 1 h, the culture was washed, adjusted to 2x10^8^ UFC/ml in EMEM and 200 µl were used to infect HEp-2 cells (100:1, *E. coli*:HEp-2). Cells were washed after 3 h and *E. coli* adhesion was determined.

c) Confluent HEp-2 cells monolayer pre-incubated with Sub-MBC antimicrobials. After 1 h, cells were washed and added bacteria (100:1, *E. coli*:HEp-2). Cells were washed after 3 h and *E. coli* adhesion ability was determined.

d) Confluent HEp-2 cells monolayer were washed and incubated with bacteria (100:1, *E. coli*:HEp-2). After 1 h cells were washed to discard bacteria non-adhered. Cells with adhere bacteria were incubated with Sub-MBC antimicrobials, and after 2 h were washed and *E. coli* adhesion ability was determined.

e) Non-adhered bacteria of assay (a) were washed and used for adhesion assay as (a). This procedure was repeated 3 times.
