# Supplementary material for: Natural and synthetic antimicrobials reduce adherence of enteroaggregative and enterohemorrhagic Escherichia coli to epithelial cells
Source: PLoS One. 2021 May 3;16(5):e0251096. doi: 10.1371/journal.pone.0251096 (PMC8092791; doi:10.1371/journal.pone.0251096)
Supplement: S1 Table — (DOCX) [file pone.0251096.s002.docx]

| S1 Table. Genes and primers used to detect the expression of virulence genes of *E. coli* strains and the expression of oxidative stress genes in HEp-2 cells, after adhesion assay. | | | | | |  |
| --- | --- | --- | --- | --- | --- | --- |
| Gene | *E. coli* Serotype | Physiological process involved | Primer or Oligonucleotide  sequence (5´-3´) | Amplicon  size (bp) | Reference |  |
| *16S rRNA* | O104:H4  O157:H7  042 Chile | 16S ribosomal RNA  (Housekeeping gene) | _F CGATGCAACGCGAAG  _R CCGGACCGCTGGCAA | 178 | [15] |  |
| *aggR* | O104:H4  042 Chile | Putative transcriptional activator *aggR* (AAF-III) regulatory protein | F_ CCGATAAGGTCAGAAACACA  R_ TGCTGCTTTGCTCATTCTTG | 169 | [15] |  |
| *pic* | O104:H4  042 Chile | SPATE | F_ CCTGACAGAGGACACGTTCA  R_TCAACCCCTGTTCTTCCAAC | 147 | [15] |  |
| *aggA* | O104:H4  042 Chile | Major protein AAF | F_CAAACAACTCAGACAATCCGCC  R_AATCAACTGCAGCATGGAGTAT | 206 | This study |  |
| *aap* | O104:H4  042 Chile | Dispersin | F_AAGTTTGTTATCTTTTCTGGCAT  R_CCCATTCGGTTAGAGCACTATA | 131 | This study |  |
| *stx* | O104:H4  O157:H7 | Shiga toxin 2a | F_GAAGATGTTTATGGCGGT  R_ CACTGTAAATGTGTCATC | 115 | [15] |  |
| *eae* | O157:H7 | Intimin adherence protein | F_CATTATGGAACGGCA  R_ACGGATATCGAAGCC | 375 | This study |  |
| *RPS18* | HEp-2 | 18S ribosomal RNA  (Housekeeping gene) | F_TGTGGTGTTGAGGAAAGCAG  R_AAGTGACGCAGCCCTCTATG | 252 | [51] |  |
| *SO1* | HEp-2 | Catalyzes the dismutation of superoxide, into oxygen and hydrogen peroxide | F_AGGGCATCATCAATTTCGAG  R_TGCCTCTCTTCATCCTTTGG | 196 | [52] |  |
| *SO2* | HEp-2 |  | F_GGAACGGGGACACTTACAAA  R_GGTAGTAAGCGTGCTCCCAC | 110 | [52] |  |
| *CAT* | HEp-2 | Hydrogen peroxide oxide reductase | F_TAAGACTGACCAGGGCATC  R_CAAACCTTGGTGAGATCGAA | 201 | [52] |  |
| *GPx* | HEp-2 | Peroxide reduction through glutathione. | F_CAACCAGTTTGGGCATCAG  R_CCCACCAGGAACTTCTCAAA | 295 | This study |  |
