# Supplementary material for: Natural and synthetic antimicrobials reduce adherence of enteroaggregative and enterohemorrhagic Escherichia coli to epithelial cells
Source: PLoS One. 2021 May 3;16(5):e0251096. doi: 10.1371/journal.pone.0251096 (PMC8092791; doi:10.1371/journal.pone.0251096)
Supplement: S2 Table — Values in parentheses indicate sub-MBC concentrations used. (DOCX) [file pone.0251096.s003.docx]

| S2 Table. Minimal bactericidal concentration (MBC, mg/ml) of natural extracts and compounds of three *E. coli* serotypes. Values in parentheses indicate sub-MBC concentrations used. | | | | | |
| --- | --- | --- | --- | --- | --- |
| Strain  *E. coli* | MBC (mg/ml) | | | | |
|  | Rifaximin | Carvacrol | Oregano | Brazilin | *Hb* |
| EAHEC, STEC  O104:H4 | 0.03 ±0.0^ab^  (0.002 & 0.005) | 0.05 ±0.0^ab^  (0.01 & 0.025) | 0.75 ±0.1^c^  (0.20 & 0.40) | 3.20 ±0.1^c^  (1.0 & 1.5) | 4.30 ±0.0^d*^  (1.5 & 3.0) |
| EHEC, STEC  O157:H7 | 0.03 ±0.0^ab^  (0.002 & 0.005) | 0.06 ±0.0^ab^  (0.01 & 0.025) | 0.85 ±0.0^c^  (0.20 & 0.40) | 2.85 ±0.1^c^  (1.0 & 1.5) | 3.80 ±0.0^c^  (1.5 & 3.0) |
| EAEC  042 Chile | 0.02 ±0.0^a^  (0.002 & 0.005) | 0.06 ±0.0^ab^  (0.01 & 0.025) | 0.70 ±0.0^c^  (0.20 & 0.40) | 2.65 ±0.1^c^  (1.0 & 1.5) | 3.80 ±0.1^c^  (1.5 & 3.0) |
| ±: Standard deviation  Different letters indicate significant differences that differ between treatments. The MBC of each antimicrobial was individually analyzed  *significant differences (*p* < 0.05) | | | | | |
