# Supplementary material for: Natural and synthetic antimicrobials reduce adherence of enteroaggregative and enterohemorrhagic Escherichia coli to epithelial cells
Source: PLoS One. 2021 May 3;16(5):e0251096. doi: 10.1371/journal.pone.0251096 (PMC8092791; doi:10.1371/journal.pone.0251096)
Supplement: S3 Table — (DOCX) [file pone.0251096.s004.docx]

| Sub-MBC | Control | Rifaximin | pH  Carvacrol | Oregano | Brazilin | *Hb* |
| --- | --- | --- | --- | --- | --- | --- |
|  | Mueller Hinton broth | | | | | |
| High | 7.4 ±0.1^b^ | 7.8 ±0.1^c*^ | 7.0 ±0.2^a*^ | 7.6 ±0.3^b^ | 7.5 ±0.1^b^ | 7.2 ±0.2^b^ |
| Low |  | 7.7 ± 0.1^c*^ | 7.5 ±0.1^b^ | 7.2 ±0.1^b^ | 7.1 ±0.1^a*^ | 7.2 ±0.1^b^ |
|  | Eagle’s Minimum Essential Medium | | | | | |
| High | 7.4 ±0.2^b^ | 8.1 ±0.1^c*^ | 7.3 ±0.1^b^ | 7.7 ±0.1^b^ | 7.8 ±0.2^c*^ | 7.3 ±0.1^b^ |
| Low |  | 7.9 ±0.1^c*^ | 7.6 ±0.1^b^ | 7.4 ±0.1^b^ | 7.5 ±0.1^b^ | 7.1 ±0.2^a*^ |

S3 Table. pH measurement of culture media with antimicrobial compounds.

±: Standard deviation.

Different letters indicate significant differences from the control. The “primary” control group was media with antimicrobials and without bacteria.

* Significant difference (*p* < 0.05)
