# Supplementary material for: Natural and synthetic antimicrobials reduce adherence of enteroaggregative and enterohemorrhagic Escherichia coli to epithelial cells
Source: PLoS One. 2021 May 3;16(5):e0251096. doi: 10.1371/journal.pone.0251096 (PMC8092791; doi:10.1371/journal.pone.0251096)
Supplement: S4 Table — (DOCX) [file pone.0251096.s005.docx]

S4 Table. Percentage of bacterial mortality after the exposure to higher sub-CMB of antimicrobials (determined by flow cytometry)

| Bacterial  strains | Exposure time (h) | Control | Rifaximin  0.005 mg/ml | Carvacrol  0.025 mg/ml | Oregano  0.40 mg/ml | Brazilin  1.5 mg/ml | *Hb*  3.0 mg/ml |
| --- | --- | --- | --- | --- | --- | --- | --- |
| EAHEC  O104:H4 | 1 | 0.2 ±0.0^a^ | 0.6 ±0.0^ab^ | 0.1 ±0.0^a^ | 0.3 ±0.1^a^ | 0.1 ±0.0^a^ | 1.0 ±0.1^a^ |
|  | 4 | 0.2 ±0.0^a^ | 0.8 ±0.1^b*^ | 0.1 ±0.0^a^ | 0.1 ±0.0^a^ | 0.1 ±0.0^a^ | 0.3 ±0.1^a^ |
| EHEC  O157:H7 | 1 | 1.4 ±0.7^a^ | 1.1 ±0.4^a^ | 1.3 ±0.5^a^ | 2.9 ±1.3^b*^ | 1.2 ±0.4^a^ | 0.8 ±0.3^a^ |
|  | 4 | 2.9 ±0.9^ab^ | 1.0 ±0.6^a^ | 1.7 ±0.7^ab^ | 3.8 ±1.4^ab^ | 4.5 ±1.3^b^ | 1.1 ±0.3^a^ |
| EAEC  042 Chile | 1 | 0.9 ±0.5^a^ | 3.3 ±0.8^c*^ | 1.2 ±0.5^a^ | 0.5 ±0.2^a^ | 2.1 ±1.1^b*^ | 1.1 ±0.7^a^ |
|  | 4 | 1.3 ±0.9^ab^ | 2.2 ±1.3^c*^ | 0.6 ±0.2^a^ | 0.7 ±0.2^a^ | 1.9 ±0.8^bc^ | 1.3 ±0.8^ab^ |

±: Standard deviation, 0.0 = ≤0.04 (standard deviation).

Different letters indicate significant differences from the control. The “primary” control group was bacteria preincubated for 1 and 4 h without antimicrobials.

* Significant difference (*p* < 0.05)
