# Supplementary material for: Natural and synthetic antimicrobials reduce adherence of enteroaggregative and enterohemorrhagic Escherichia coli to epithelial cells
Source: PLoS One. 2021 May 3;16(5):e0251096. doi: 10.1371/journal.pone.0251096 (PMC8092791; doi:10.1371/journal.pone.0251096)
Supplement: S5 Table — (DOCX) [file pone.0251096.s006.docx]

S5 Table. Percentage of HEp-2 cells viability by MTT method determination after exposure to sub-CMB of antimicrobials (mg/ml).

| Antimicrobials | Sub-CMB  mg/ml | Exposure time (hours) | | |
| --- | --- | --- | --- | --- |
|  |  | 4 | 12 | 24 |
| Rifaximina | 0.005  0.002 | 110.5 ±1.7^bc^  97.1±1.1^ab^ | 104.3 ±2.4^b^  105.2 ±1.9^b^ | 99.7 ±0.4^b^  100.9 ±1.2^b^ |
| Carvacrol | 0.025  0.010 | 70.1 ±0.8ª*  91.3 ±2.7^ab^ | 64.8 ±1.3^a*^  83.6 ±0.5^ab^ | 37.5 ±0.3^a*^  82.2 ±1.0^ab^ |
| Oregano extract | 0.40  0.20 | 95.4 ±2.1^ab^  96.9 ±1.6^ab^ | 85.6 ±0.4^ab^  87.1 ±0.7^ab^ | 84.1 ±1.8^ab^  87.9 ±1.3^ab^ |
| Brazilin | 1.5  1.0 | 100.8 ±0.7^b^  108.4 ±1.1^b^ | 107.6 ±1.5^b^  98.4 ±0.6^b^ | 105.1 ±0.3^b^  99.0 ±2.2^b^ |
| Hb extract | 3.0  1.5 | 103.5 ±0.43^b^  109.6 ±1.8^b^ | 96.9 ±1.0^ab^  98.1 ±0.9b | 95.3 ±1.2^ab^  94.8 ±1.7^ab^ |

±: Standard deviation

Different letters indicate significant differences from the control. The “primary” control group was HEp-2 cells incubated for 4, 12 and 24 h without antimicrobials.

* Significant difference (p < 0.05)
