# Supplementary material for: Natural and synthetic antimicrobials reduce adherence of enteroaggregative and enterohemorrhagic Escherichia coli to epithelial cells
Source: PLoS One. 2021 May 3;16(5):e0251096. doi: 10.1371/journal.pone.0251096 (PMC8092791; doi:10.1371/journal.pone.0251096)
Supplement: S6 Table — (DOCX) [file pone.0251096.s007.docx]

S6 Table. Number of bacteria and bacterial aggregates adhered to HEp-2 cells stained by GIEMSA.

| Compound/  extract | (mg/ml) | Count / field (x̄) | |  |
| --- | --- | --- | --- | --- |
|  |  | Adhered bacteria  (alone) | Adhered bacteria (aggregates) | |
| Control |  | 52 ±9.6 | 5 ± 1.8 ^+++^  27 ± 4.4 ^++^  32 ± 3.7 ^+^ | |
| Rifaximin | 0.005 | 22 ±6.3 | 18 ±2.4 ^++^  7 ±2.0 ^+^ | |
| Carvacrol | 0.10 | 158 ±10.8 | 2 ±0.8 ^++^  5 ±1.3 ^+^ | |
| Oregano Extract | 0.40 | 41 ±7.1 | 23 ± 3.2 ^++^  12 ±2.8 ^+^ | |

Bacterial aggregates formed by ≤10 bacteria (+), between 10 and 50 bacteria (++), ≥50 bacteria (+++).
