# Supplementary material for: Natural and synthetic antimicrobials reduce adherence of enteroaggregative and enterohemorrhagic Escherichia coli to epithelial cells
Source: PLoS One. 2021 May 3;16(5):e0251096. doi: 10.1371/journal.pone.0251096 (PMC8092791; doi:10.1371/journal.pone.0251096)
Supplement: Attachment — Submitted filename: Response to Reviewers.pdf [file pone.0251096.s009.pdf]

PLOS ONE
Attn: Dr. Yung-Fu Chang
Academic Editor
E-mail: em@editorialmanager.com

Re: Response to reviewer comments for PONE-D-20-36066

Dear Dr. Chang:

Thank you for comments to PONE-D-20-36066, "Natural and synthetic antimicrobials reduce adherence of enteroaggregative and enterohemorrhagic Escherichia coli to epithelial cells". We detail below an itemized numbered list stating (in italics) how we addressed each of the reviewers' concerns in a revised manuscript.

1.) Please ensure that your manuscript meets PLOS ONE's style requirements, including those for file naming. The PLOS ONE style templates can be found at

https://journals.plos.org/plosone/s/file?id=wjVg/PLOSOne_formatting_sample_main_body.pdf and https://journals.plos.org/plosone/s/file?id=ba62/PLOSOne_formatting_sample_title_authors_affiliations.pdf
R= MS was checked according to the journal instructions

2.) We note that you have included the phrase "data not shown" in your manuscript. Unfortunately, this does not meet our data sharing requirements. PLOS does not permit references to inaccessible data. We require that authors provide all relevant data within the paper, Supporting Information files, or in an acceptable, public repository. Please add a citation to support this phrase or upload the data that corresponds with these findings to a stable repository (such as Figshare or Dryad) and provide and URLs, DOIs, or accession numbers that may be used to access these data. Or, if the data are not a core part of the research being presented in your study, we ask that you remove the phrase that refers to these data.
R= The legend was deleted since this finding was not relevant to this research

3.)PLOS ONE now requires that authors provide the original uncropped and unadjusted images underlying all blot or gel results reported in a submission's figures or Supporting Information files. This policy and the journal's other requirements for blot/gel reporting and figure preparation are described in detail at https://journals.plos.org/plosone/s/figures#loc-blot-and-gel-reporting-requirements and https://journals.plos.org/plosone/s/figures#loc-preparing-figures-from-image-files. When you submit your revised manuscript, please ensure that your figures adhere fully to these guidelines and provide the original underlying images for all blot or gel data reported in your submission. See the following link for instructions on providing the original image data: https://journals.plos.org/plosone/s/figures#loc-original-images-for-blots-and-gels.
R= Image files were not manipulated or adjusted in any way. Pictures were taken directly from microscope.

In your cover letter, please note whether your blot/gel image data are in Supporting Information or posted at a public data repository, provide the repository URL if relevant, and provide specific details as to which raw blot/gel images, if any, are not available. Email us at plosone@plos.org if you have any questions.

4.) Please upload a new copy of Figure 1 as the detail is not clear. Please follow the link for more information: https://blogs.plos.org/plos/2019/06/looking-good-tips-for-creating-your-plos-figures-graphics/" https://blogs.plos.org/plos/2019/06/looking-good-tips-for-creating-your-plos-figures-graphics/
R= An improved fig 1 was included.

Reviewer #1 (Remarks to Author):

Reviewer #1: The overall study is good. However, the way the manuscript is written is a bit confusing at several places. Consider reframing some sections.
Reference 1 is not in English
R= Done

Line 41 reducing up to to 65% the percentage of E.coli adhered
R= the words *"the percentage"* were deleted in text.

Line 222- The culture medium was changed when it changed color (how often was that? please explain why)
R= Media was changed at 48-72 h. Media changed of color due to acidification of pH indicator as consequence of cellular growth. *This information was included in the text.*

Line 281- A 200 ul aliquot
R= Done

Reference where in the manuscript is the data for the actual numbers that were counted after the incubation mentioned below.
Line 233- The number of
234 adhered bacteria was determined by adding 200 µl of 0.5% sodium deoxycholate
235 (Sigma-Aldrich) to detach the bacteria from the cells. Detached bacteria were 10-
236 fold serially diluted, plated on MH agar, and counted after incubation at 37°C for
237 48 h. The adherence pattern was determined by Giemsa staining. Samples of
238 adhered and non-adhered bacteria were collected for gene expression assays.
R= Data appear in Figure 1 which was improved.

The oregano extracts can not be consistent every time when prepared. What do the authors do the make sure the extracts are not too different each time prepared?

R= The same batch of oregano was always used. It was preserved in dry leaves, and under controlled conditions of light (darkness), humidity and temperature. When the fresh extract was prepared, the MBC (minimum bactericidal concentration) was determined; no variations on MBC was found. This information was included in the text.

Sincerely Yours:

Norma Laura Heredia

Norma L. Heredia
