# Supplementary material for: Natural and synthetic antimicrobials reduce adherence of enteroaggregative and enterohemorrhagic Escherichia coli to epithelial cells
Source: PLoS One. 2021 May 3;16(5):e0251096. doi: 10.1371/journal.pone.0251096 (PMC8092791; doi:10.1371/journal.pone.0251096)
Supplement: Attachment — Submitted filename: Response to Reviewers.pdf [file pone.0251096.s010.pdf]

UANL
UNIVERSIDAD AUTÓNOMA DE NUEVO LEÓN
FACULTAD DE CIENCIAS BIOLÓGICAS
LABORATORIO DE BIOQUÍMICA Y GENÉTICA DE MICROORGANISMOS

PLOS ONE
Attn: Dr. Yung-Fu Chang
Academic Editor
E-mail: em@editorialmanager.com

Re: Response to reviewer comments for PONE-D-20-36066R1

Dear Dr. Chang:

Thank you for comments to PONE-D-20-36066R1, "Natural and synthetic antimicrobials reduce adherence of enteroaggregative and enterohemorrhagic Escherichia coli to epithelial cells". We detail below an itemized numbered list stating (in italics) how we addressed each of the reviewers' concerns in a revised manuscript.

Please review your reference list to ensure that it is complete and correct. If you have cited papers that have been retracted, please include the rationale for doing so in the manuscript text, or remove these references and replace them with relevant current references. Any changes to the reference list should be mentioned in the rebuttal letter that accompanies your revised manuscript. If you need to cite a retracted article, indicate the article's retracted status in the References list and also include a citation and full reference for the retraction notice.
R= All references were checked. No cited paper has been retracted

Reviewer #1 (Remarks to Author):
Comments to the Author

The authors need to check the italics on gene names. It isn't consistent throughout the manuscript.
R= In this manuscript we referred to Shiga toxin 1 protein and/or Shiga toxin 2 protein as Stx1 and Stx2 respectively (these are not genes). We slightly modified the text to clarify this.

Sincerely Yours:
Norma L. Heredia

Av. Pedro de Alba y Manuel L. Barragán *sIn,* Cd. Universitaria, C.P. 66455, San Nicolás de los Garza, Nuevo León, México.
Tels. +52 818376 3044 • 8329 4000, ext. 6466. www.microbiosymas.com
