# Supplementary material for: Natural and synthetic antimicrobials reduce adherence of enteroaggregative and enterohemorrhagic Escherichia coli to epithelial cells
Source: PLoS One. 2021 May 3;16(5):e0251096. doi: 10.1371/journal.pone.0251096 (PMC8092791; doi:10.1371/journal.pone.0251096)
Supplement: Attachment — Submitted filename: plosone.docx [file pone.0251096.s008.docx]

The overall study is good. However, the way the manuscript is written is a bit confusing at several places. Consider reframing some sections.

Reference 1 is not in English

Line 41 reducing up to to 65% the percentage of *E.coli* adhered

Line 222- The culture medium was changed when it changed color (how often was that? please explain why)

Line 281- A 200 ul aliquot

Reference where in the manuscript is the data for the actual numbers that were counted after the incubation mentioned below.

Line 233- The number of

234 adhered bacteria was determined by adding 200 μl of 0.5% sodium deoxycholate

235 (Sigma-Aldrich) to detach the bacteria from the cells. Detached bacteria were 10-

236 fold serially diluted, plated on MH agar, and counted after incubation at 37°C for

237 48 h. The adherence pattern was determined by Giemsa staining. Samples of

238 adhered and non-adhered bacteria were collected for gene expression assays.

The oregano extracts can not be consistent every time when prepared. What do the authors do the make sure the extracts are not too different each time prepared?
